# A comparison of bayesian adaptive randomization and multi-stage designs for multi-arm clinical trials

**DOI:** 10.1186/1745-6215-14-S1-P40

**Published:** 2013-11-29

**Authors:** James Wason, Lorenzo Trippa

**Affiliations:** 1 MRC Biostatistics Unit, Cambridge, UK; 2 Harvard School of Public Health, Boston, USA

## 

When several experimental treatments are available for testing, multi-arm trials provide gains in efficiency over separate trials. Including interim analyses allows the investigator to effectively use the data gathered during the trial. Bayesian adaptive randomization (AR) and multi-arm multi-stage (MAMS) designs are two distinct methods that use patient outcomes to improve the efficiency and ethics of the trial. AR allocates a greater proportion of future patients to treatments that have performed well; MAMS designs use pre-specified stopping boundaries to determine whether experimental treatments should be dropped. There is little consensus on which method is more suitable for clinical trials. In this presentation we compare the two designs under several simulation scenarios and in the context of a real multi-arm phase II breast cancer trial. We compare the methods in terms of their efficiency and ethical properties. The practical problem of a delay between recruitment of patients and assessment of their treatment response is also considered. Both methods are more efficient and ethical than a multi-arm trial without interim analyses. Delay between recruitment and response assessment attenuates this efficiency gain. Our comparisons show that AR is more efficient than MAMS designs when there is an effective experimental treatment; while if none of the experimental treatments is effective, then MAMS designs slightly outperform AR.

